# Virtual Coronary Intervention: A Treatment Planning Tool Based Upon the Angiogram

**DOI:** 10.1016/j.jcmg.2018.01.019

**Published:** 2019-05

**Authors:** Rebecca C. Gosling, Paul D. Morris, Daniel A. Silva Soto, Patricia V. Lawford, D. Rodney Hose, Julian P. Gunn

**Affiliations:** a Department of Infection, Immunity and Cardiovascular Disease, University of Sheffield, Sheffield, United Kingdom; b Department of Cardiology, Sheffield Teaching Hospitals, National Health Service Foundation Trust, Northern General Hospital, Sheffield, United Kingdom; c Insigneo Institute for In Silico Medicine, Sheffield, United Kingdom; d Department of Circulation and Medical Imaging, Norwegian University of Science and Technology (NTNU), Trondheim, Norway

**Keywords:** computational fluid dynamics, coronary artery disease, coronary physiology, fractional flow reserve, percutaneous coronary intervention, CFD, computational fluid dynamics, CTA, computed tomography angiography, FFR, fractional flow reserve, mFFR, measured fractional flow reserve, PCI, percutaneous coronary intervention, VCI, virtual coronary intervention, vFFR, virtual fractional flow reserve

## Abstract

**Objectives:**

This study sought to assess the ability of a novel virtual coronary intervention (VCI) tool based on invasive angiography to predict the patient’s physiological response to stenting.

**Background:**

Fractional flow reserve (FFR)-guided percutaneous coronary intervention (PCI) is associated with improved clinical and economic outcomes compared with angiographic guidance alone. Virtual (v)FFR can be calculated based upon a 3-dimensional (3D) reconstruction of the coronary anatomy from the angiogram, using computational fluid dynamics (CFD) modeling. This technology can be used to perform virtual stenting, with a predicted post-PCI FFR, and the prospect of optimized treatment planning.

**Methods:**

Patients undergoing elective PCI had pressure-wire–based FFR measurements pre- and post-PCI. A 3D reconstruction of the diseased artery was generated from the angiogram and imported into the VIRTUheart workflow, without the need for any invasive physiological measurements. VCI was performed using a radius correction tool replicating the dimensions of the stent deployed during PCI. Virtual FFR (vFFR) was calculated pre- and post-VCI, using CFD analysis. vFFR pre- and post-VCI were compared with measured (m)FFR pre- and post-PCI, respectively.

**Results:**

Fifty-four patients and 59 vessels underwent PCI. The mFFR and vFFR pre-PCI were 0.66 ± 0.14 and 0.68 ± 0.13, respectively. Pre-PCI vFFR deviated from mFFR by ±0.05 (mean Δ = −0.02; SD = 0.07). The mean mFFR and vFFR post-PCI/VCI were 0.90 ± 0.05 and 0.92 ± 0.05, respectively. Post-VCI vFFR deviated from post-PCI mFFR by ±0.02 (mean Δ = −0.01; SD = 0.03). Mean CFD processing time was 95 s per case.

**Conclusions:**

The authors have developed a novel VCI tool, based upon the angiogram, that predicts the physiological response to stenting with a high degree of accuracy.

## Graphical abstract

**Figure:**
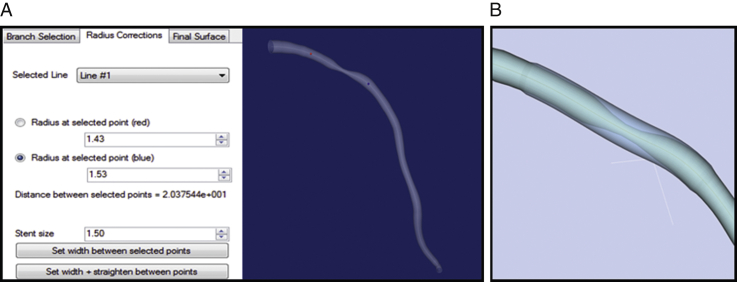

Percutaneous coronary intervention (PCI) guided by fractional flow reserve (FFR) is superior to angiography alone, with improved clinical and economic outcomes 1, 2. However, it is currently used in few patients because it is invasive and time consuming and requires pharmacological induction of hyperemia (3). Using computational fluid dynamics (CFD) modeling, it is possible to calculate a virtual FFR (vFFR) from a 3-dimensional (3D) reconstruction of the coronary angiogram without the need for invasive pressure wire measurements. This imaging-based solution predicts invasively measured fractional flow reserve (mFFR) with a high level of accuracy (4). We have developed virtual coronary intervention (VCI) as an extension to this technology. VCI allows an idealized virtual stent(s) to be inserted and the vFFR to be recalculated. The ability to predict the physiological response to a variety of potential stenting strategies would be advantageous in terms of interventional planning.

The aim of this project, therefore, was to develop and validate a system capable of predicting the physiological response to a planned PCI based solely upon coronary angiographic images.

## Methods

### Study design

This was a single-site cohort study carried out at the Northern General Hospital, Sheffield, United Kingdom, which is a tertiary cardiac center. The study protocol was approved by the local ethics committee (13/YH/0070).

### Study population

Data were collected prospectively for patients undergoing elective PCI between 2014 and 2016. Consecutive patients 18 years of age and older who had angiographically confirmed coronary disease (30% to 90% stenosis by visual angiographic assessment) were recruited. Patients were excluded if they had presented acutely within the previous 60 days, had prior coronary artery bypass graft surgery, had chronic total occlusion(s), if passage of a pressure wire would be unsafe, or if the patient was unable or unwilling to consent. Written informed consent was obtained from all participating patients. Clinical, demographic, FFR, and angiographic data were collected prospectively. If patients did not proceed to PCI, either due to a negative FFR or operator judgement, they were not included. A study flow diagram is shown in Supplemental Figure 1.

### Procedure protocol

Patients underwent invasive coronary angiography using standard techniques. All arteries with disease affecting >50% vessel diameter, as determined visually, were assessed using a pressure wire (Volcano, Philips, Amsterdam, the Netherlands). Hyperemia was induced by an intravenous infusion of adenosine, 140 μg/kg/min. The FFR value was measured during stable hyperemia. The decision to proceed to PCI was made by the operator, using the findings from angiographic and invasive FFR assessments. The PCI procedure, including determining the number and sizes of stents, followed standard practice. Following PCI, a repeat FFR measurement was recorded.

### 3D reconstruction

A 3D reconstruction of the coronary anatomy was created offline at the end of the procedure using a Philips 3D workstation. Two clear orthogonal planes from similar phases of the cardiac cycle, as close to 90° apart as possible, were selected to segment and reconstruct coronary arterial geometry. The electrocardiography trace was imported alongside the angiographic images, allowing images from end-diastole to be selected. The 3D reconstruction was exported from the workstation as a virtual reality modeling language (i.e., *vrml) file to our VIRTUheart workflow (4).

### Simulated stent placement and vFFR calculation

**Figure 1:**
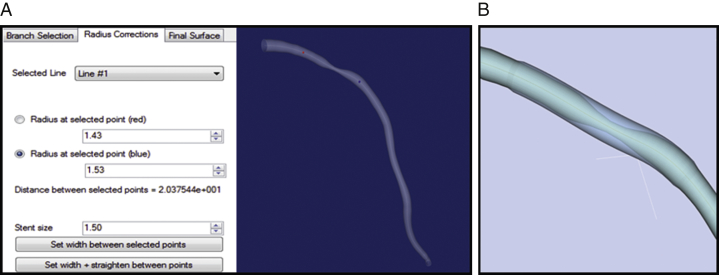
Simulated Stent Placement Within the VIRTUheart System **(A)** The 3-dimensional reconstruction of the artery is displayed on the screen, and the operators mark the arterial location where they wish to deploy a stent identified by the **red** (proximal) and **blue** (distal) markers. In the text **(left)**, the vessel radii at both selected points are displayed as well as the distance between them. The operator can adjust the radius of the desired virtual stent in the box below (“stent size”). The length can be altered by moving the position of the **redandblue dots**. In the example shown, a 3.0- × 20-mm virtual stent has been inserted by the operator. The surface mesh is manipulated to match these stenting criteria. **(B)** The new surface (the virtually stented artery) is shown overlaying the original vessel **(right panel)**.

The simulated stent placement was carried out offline within the VIRTUheart workflow environment, which replicated the dimensions and position of the stent(s) used during the PCI procedure. The geometry of the patient vessel is expressed as a set of connected circular cross sections, following the points formed in the center of the vessel path. Using the dedicated VIRTUheart graphical user interface, the operator marks the arterial location where they wish to deploy a stent (Figure 1A). The operator then determines the diameter and length of the stent they wish to deploy, just as they would in the cardiac catheter laboratory. Vessel–stent interaction is simulated by smoothing the vessel trajectory, using a cubic spline and adjusting the cross-sectional radius. The VIRTUheart software then outputs the corrected surface mesh; the virtually stented artery (Figure 1B). The final vessel geometry is composed of triangle strips connecting each cross-section, each strip containing 128 triangles. This step can be repeated if more than 1 virtual stent is to be inserted in the same artery. This permits the modeling of multiple stent strategies. The ultimate aim of this work will be that operators can compare the physiological impact of different stenting strategies before they treat a patient, so they can select the optimum approach. However, for this validation study, we compared the computed physiological result with the actual physiological result. It was therefore critical that we based the CFD simulation upon matching the virtual stent to the stent actually deployed in the cardiac catheterization laboratory.

A new surface mesh of the altered geometry was created which was then discretized (meshed) into ∼1 million tetrahedra with boundary inflation layers, prior to CFD simulation. The boundary conditions for the simulation were then defined. Boundary conditions represent the physiological conditions at each of the boundaries (ends) of the 3D domain (reconstructed vessel). The inlet boundary was set to match the mean proximal (aortic) pressure (Pa), taken from the catheter tip, data which are freely available during any routine (PCI or diagnostic) coronary catheterization procedure. The distal boundary condition is more challenging because, in the absence of passing a pressure wire, the intracoronary physiology is unknown. Therefore, we applied a generic resistance value (8.721E+9 Pa/m^3^s^−1^) obtained as an average of measured myocardial resistance from a previously studied cohort (4). vFFR was computed pre- and post-simulated stent placement using commercial CFD software (CFX, AnSys, Cannonsburg, Pennsylvania). The CFD software solves the steady-state equations of fluid flow (Navier-Stokes and continuity), in 3D, using the conservation form of the finite volume method.

### Statistical analysis

Data are presented as mean ± SD or as percentage (proportion), unless stated otherwise. mFFR and vFFR values were compared pre- and post-PCI and post-VCI. The diagnostic accuracy (the ability of VFFR to predict whether mFFR was < or >0.80) was assessed by calculating the sensitivity, specificity, positive predictive value, negative predictive value, and overall accuracy. The agreement between mFFR and vFFR was assessed using a Bland-Altman plot. Statistics were calculated using SPSS version 24 (IBM Inc., Armonk, New York).

## Results

### Patient and lesion characteristics

A total of 101 patients with angiographically confirmed disease were studied. Of these, 61 patients had a positive FFR and underwent PCI to at least 1 vessel. In 4 patients, no FFR was recorded after PCI; in 1 patient an error occurred in the recorded electrocardiography trace (so the vessel could not be segmented); and in 2 patients, the quality of the imaging was not adequate to allow satisfactory segmentation. Therefore, 54 patients were included in the final analysis. Baseline patient and lesion characteristics are shown in Table 1. Patients’ mean age was 63.2 ± 10.7; 45 patients (83%) were male; 12 patients (22%) had type 2 diabetes mellitus; and 23 patients (43%) had had a previous myocardial infarction. Five patients (9%) had multivessel PCI. In total, 59 vessels were treated (31 were left anterior descending, 7 were left circumflex, and 21 were right coronary arteries). One patient had no pre-PCI FFR because we were unable to pass the wire, giving 58 paired pre-PCI datasets (Supplemental Figure 2). In 1 case, 2 stents were inserted sequentially with an FFR measurement taken after each, giving 60 paired post-PCI datasets (Supplemental Table 1). Of the 59 vessels treated, the number of stents per vessel was 1.1 ± 0.3. The stent length and width were 24.6 ± 9.2 and 3.1 ± 0.5 mm, respectively. All patients received second-generation drug eluting stents.

**Table 1:** Baseline Patient and Lesion Characteristics

Patient characteristics	
Age, yrs	63.2 ± 10.7
Male	45 (83)
Current smoker	6 (11)
Hypertension	36 (67)
Hyperlipidemia	38 (70)
Type 2 diabetes mellitus	12 (22)
Previous MI	23 (43)
BMI, kg/m^2^	29.1 ± 3.9
Lesion characteristics	
Lesion length, mm	20.6 ± 14.4
% diameter stenosis	58 ± 13.1
Stent length, mm	24.6 ± 9.2
Stent width, mm	3.1 ± 0.5
Bifurcation disease	15 (28)
Tandem lesions	18 (33)

Values are mean ± SD or n (%).

BMI = body mass index; MI = myocardial infarction.

### Accuracy of vFFR to predict FFR pre-PCI

**Figure 2:**
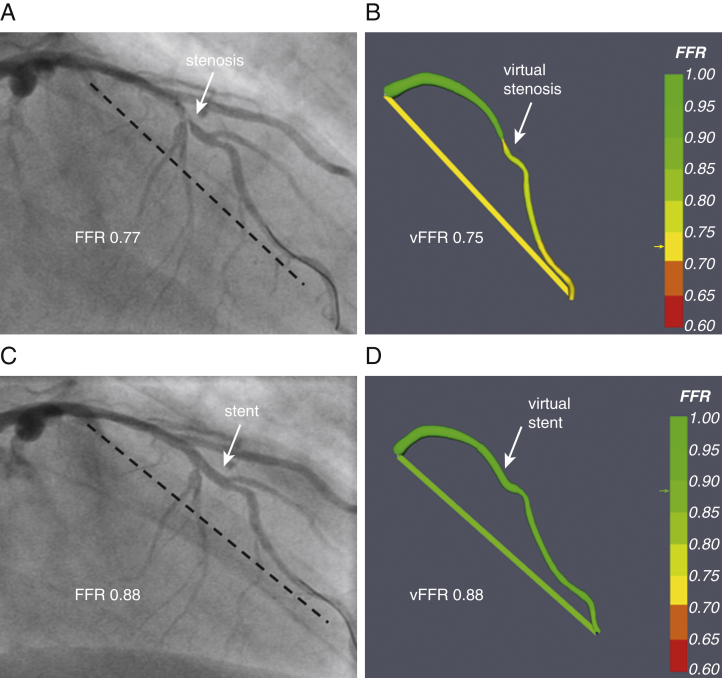
Example of VCI **(A)** A 66-year-old man presented with chronic stable angina. The LAD had a severe mid vessel stenosis **(arrow)**. The mFFR between the proximal and distal points **(dashed line)** was 0.77. **(B)** Angiograms were used to model the vFFR by using the VIRTUheart system, which was calculated to be 0.75 over the same segment. This is displayed in false color **yellow**, the **straight yellow line** connecting the same 2 points between which the vFFR was calculated, exactly matching the 2 spots marked by the **dashed line (A)**. **(C)** After implantation of a 2.75- × 18-mm stent at the stenosis, the mFFR was 0.88 over the same segment. **(D)** VCI using the VIRTUheart system was then used to implant a virtual 2.75- × 18-mm stent, and the recalculated vFFR was 0.88, corresponding to the **green line** connecting the 2 points. LAD = left anterior descending; mFFR = measured fractional flow reserve; VCI = virtual coronary intervention; vFFR = virtual FFR.

**Figure 3:**
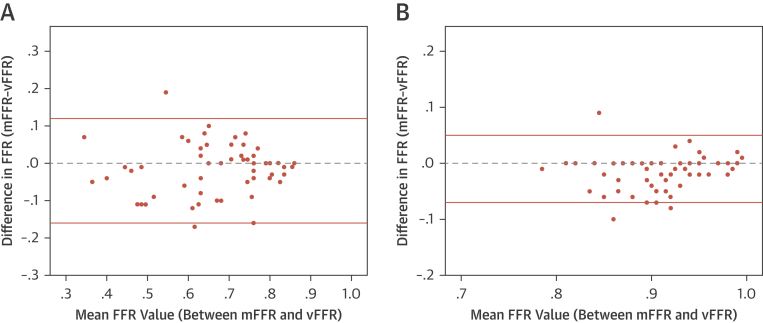
Bland Altman Plots **(A)** These figures demonstrate the differences between mFFR and vFFR plotted against the mean value pre-PCI and **(B)** post-PCI and VCI. The 2 **pink lines** represent the limits of agreement 2 SD above and below the mean delta. PCI = percutaneous coronary intervention; other abbreviations as in Figure 2.

**Figure 4:**
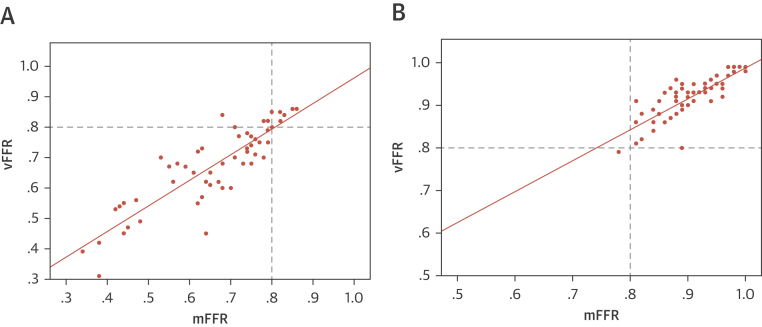
Correlation Between vFFR and mFFR **(A)** Correlation between virtual vFFR and mFFR pre-PCI and **(B)** post PCI and VCI with a line of best fit passing through the origin. R = 0.87 and 0.80, respectively. Abbreviations as in Figure 2.

CFD solutions were successfully obtained in all vessels. The CFD computational time was approximately 95 s per case (Figure 2) (5). Prior to PCI, the mean mFFR was 0.66 ± 0.14, and the mean vFFR was 0.68 ± 0.13. The mean difference (bias) between mFFR and vFFR was −0.02 ± 0.07. The average error was ±0.05 (±5%). A Bland-Altman plot is shown in Figure 3A. The vFFR and mFFR were closely correlated (r = 0.87) (Figure 4A). The diagnostic ability of vFFR to predict ischemia accurately (invasive FFR ≤0.80) was 93% (positive predictive value of 100%; negative predictive value of 64%; sensitivity of 92%; specificity of 100%).

### Accuracy of vFFR post-VCI to predict FFR post-PCI

After PCI, the mean mFFR was 0.90 ± 0.05, and the mean vFFR was 0.92 ± 0.05. The mean difference (bias) between post-PCI mFFR and post VCI-vFFR was 0.01 ± 0.03. The average absolute error was ±0.02 (±3%). A Bland-Altman plot is shown in Figure 3B. The vFFR and mFFR were closely correlated (r = 0.80) (Figure 4B).

## Discussion

We have demonstrated, for the first time, that simulated stent placement based upon invasive coronary angiography imaging is feasible and can predict the physiological results of stenting with high accuracy. Our model can produce results within minutes, in a similar (or shorter) timeframe than that required for an invasive FFR, making it suitable for use within the cardiac catheterization laboratory. These methods will allow operators to plan an optimal PCI strategy, before treatment is delivered, on a patient-specific basis.

### Advantages of VCI based upon the angiogram

Simulated stent placement has recently been demonstrated with coronary computed tomography angiography (CTA) imaging (6). Kim et al. (6) demonstrated the ability to predict residual ischemia after stenting with this technique in a small cohort of 44 patients. However, coronary CTA is still limited by heart rate control and inaccuracy in assessing calcific disease 7, 8, and the resolution is still inferior to invasive angiography. Furthermore, patients with significant disease will invariably have invasive angiographic images taken prior to PCI. VCI, particularly if available immediately, will therefore permit accurate and objective planning of complex interventions compared with operator-based predictions of the response to a particular stent strategy; particularly useful in complex disease. Although instantaneous wave-free ratio has been used to predict the response to stenting (9), this method still requires passage of a pressure wire, whereas our method is quick, easy, noninvasive, and can be done either with the patient on the table or offline after the procedure. The latter would permit assessment of angiograms that have be performed in hospitals that do not have access to pressure wire technology.

### VCI for optimization of PCI

FFR measurement after stenting has been shown to predict adverse events at follow-up. Increased rates of major adverse cardiac events at 6 months and 1 year have been demonstrated in patients with a post-procedural FFR <0.90 10, 11. The ability to predict the physiological outcome of a number of alternative stenting strategies would permit the operator to identify the optimal approach prior to intervention. The primary aim of this study was to validate the accuracy of the computed results, a critical first step. Our tool permits multiple stenting strategies to be simulated, and the physiological results of each strategy to be compared, thus facilitating the selection of the best PCI strategy before proceeding with intervention. Currently, each simulation takes approximately 95 s, and the cumulative time is dependent upon the number of strategies being compared. However, as we develop this tool further, we aim to implement computational methods which significantly accelerate processing time, enabling very rapid CFD results for each strategy, with minimal time cost to the clinician. In addition to prediction of the physiological results associated with virtual PCI, the tool may also facilitate the selection of the ideal stent diameter and length because the graphical user interface reports the diameter along the artery at all points (stenotic and reference segments) and the length between user-specified points. Although this is not the primary aim of this tool, these 3D data, based upon the reconstructed artery prior to VCI, may add supplementary data useful to the operator (Figure 1A). For a simple case, such as an isolated severe stenosis with an appropriate clinical background, the use of VCI technology is unnecessary, but in cases with serial lesions (12), diffuse disease, or bifurcations, it may have value. This could increase the likelihood of achieving an optimal post-treatment FFR, potentially improving outcomes. Specifically, it will be able to predict the maximum realistically achievable FFR in the context of other disease. It may indeed reveal that localized stenting in a diffusely diseased vessel is pointless. On the other hand, it may show that a modest increase in length or width of a stent could provide a substantially improved final FFR. Clinical judgment will always be required, because absolute optimization of the post-PCI FFR with excessively long and wide stents would be both unrealistic and hazardous in the real world.

### VCI to assess tandem lesions

**Figure 5:**
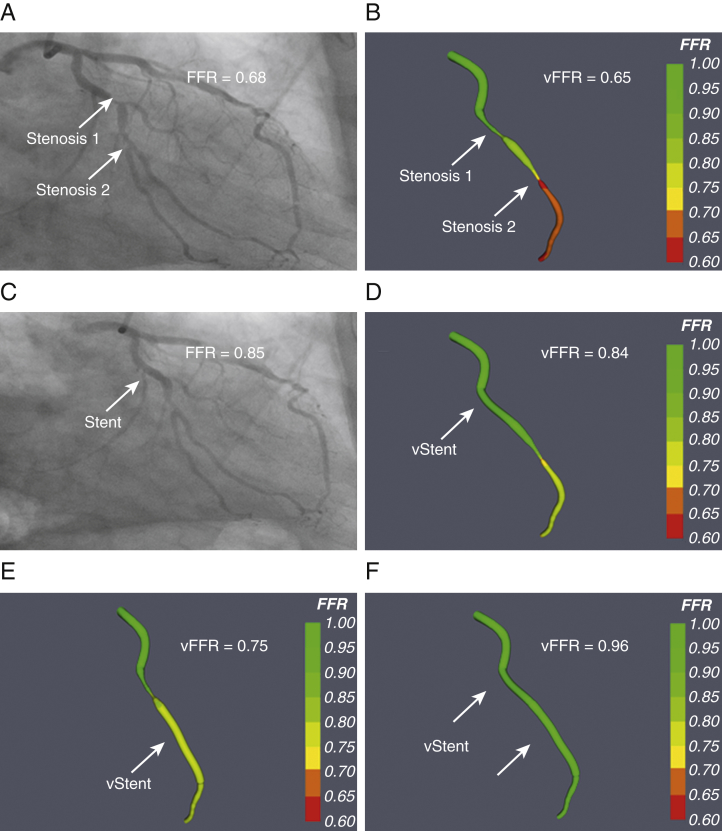
Using VCI To Assess the Functional Significance of Tandem Lesions A 67-year-old man presented with chronic stable angina. There were tandem lesions in the left circumflex artery. **(A)** The measured FFR at the distal vessel was 0.68. **(B)** The angiogram was used to model the vFFR, which was 0.65 over the same segment of artery. **(C)** PCI was undertaken to the proximal lesion with implantation of a 3.5- × 12-mm drug-eluting stent, and the post-procedural FFR was 0.85. The distal lesion was left untreated. VCI was used to assess the lesions individually. The proximal stenosis was removed by inserting a 3.5- × 12-mm virtual stent. **(D)** The recalculated vFFR at the distal vessel was 0.84. **(E)** The distal stenosis was removed by inserting a 2.75- × 20-mm virtual stent, and the recalculated vFFR at the distal vessel was 0.75. **(F)** Following VCI to both stenoses in sequence, the vFFR was 0.96. Abbreviations as in Figures 2 and 3.

In the presence of tandem or serial lesions, it is impossible to determine accurately the impact of each individual lesion upon coronary blood flow by using invasive pressure wire assessment. A distal stenosis provides a fixed resistor which is not amenable to vasodilation, so assessment of a proximal lesion underestimates its functional significance (12). Only by removing a stenosis (physically or with our system, virtually) is it possible to increase hyperemic flow. This is often the strategy used in FFR-guided PCI, whereby the operator will stent the lesion believed to contribute most to the aggregate FFR, whether based upon a pullback, with all its flaws, or not. This may lead to the unnecessary stenting of one or other lesion. Some groups have proposed methods of calculating the “true” FFR from the acquired values. However, many of these methods require the measurement of the coronary wedge pressure which can only be obtained during balloon coronary occlusion (12). In contrast, by using our VCI tool, the operator can “remove” each stenosis in turn to assess the true impact of each individual lesion. An example of our VCI tool being used to assess tandem lesions in this way is shown in Figure 5. Further outcome studies evaluating this approach are warranted.

### Study limitations

The number of cases used in this proof-of-concept study is modest and performed under ideal circumstances in elective cases. Further studies in patients with complex disease and acute cases are warranted before these results can be extrapolated. We used a generic distal boundary condition in our model, and this does not provide the level of accuracy as applying personalized distal boundary conditions. However, personalized tuning requires invasive measurements from a sensor-tipped angioplasty wire, and the aim of this study was to assess methods that did not require invasive instrumentation. Although the use of a generic distal boundary condition has previously been shown to be associated with accurate results, it may be less accurate in patients with significantly abnormal myocardial resistance, such as in patients with myocardial infarction or left ventricular hypertrophy 5, 13. Also, our CFD analysis was based upon a single lumen reconstruction; side branches were not included. This may result in an overestimation of the pressure drop. However, despite this limitation, our model predicted invasive FFR with high accuracy. Also, it was assumed that adequate stent deployment was achieved. VCI predicts the physiological response to stenting and is not intended to be a replacement for intravascular ultrasonography or optical coherence tomography in determining procedural success, which is dependent upon other procedural factors. Finally, this was a proof-of-concept study, and further work is required to demonstrate the clinical utility of the VCI tool in a prospective trial.

## Conclusions

The authors have demonstrated the ability of VCI, based upon the invasive angiogram, to predict the physiological response to stenting with a high degree of accuracy. FFR post-PCI was reliably predicted, in a cohort of stable elective patients, without requiring passage of pressure wire or pharmacological induction of hyperemia. Computational time was 95 s per case making it suitable for use within the cardiac catheterization laboratory. This novel image-based technique could lead to accurate, patient-specific, revascularization planning.

Perspectives **COMPETENCY IN MEDICAL KNOWLEDGE:** VCI based upon invasive angiographic imaging is feasible and can predict physiological response to stenting with high accuracy. The processing time is short, making it practical for use as a treatment planning tool.

**TRANSLATIONAL OUTLOOK:** Angiography-based VCI requires further study in the assessment of complex disease, and larger outcome studies are warranted.
