# Supplementary material for: Virtual Coronary Intervention: A Treatment Planning Tool Based Upon the Angiogram
Source: JACC Cardiovasc Imaging. 2019 May;12(5):865–72. doi: 10.1016/j.jcmg.2018.01.019 (PMC6503181; doi:10.1016/j.jcmg.2018.01.019)
Supplement: Supplemental Figures 1 and 2 and Supplemental Table 1 [file mmc1.docx]

**Online Figure 1: Flow diagram of study recruitment**

One hundred and one patients with angiographically confirmed disease were studied. Of these, 61 had a positive FFR and underwent PCI to at least one vessel. In four patients, there was no FFR recorded after PCI, in one patient there was an error in the recorded ECG trace (so the vessel could not be segmented), and in two cases the quality of the imaging was not adequate to allow satisfactory segmentation. Therefore 54 patients were included in the final analysis.

**
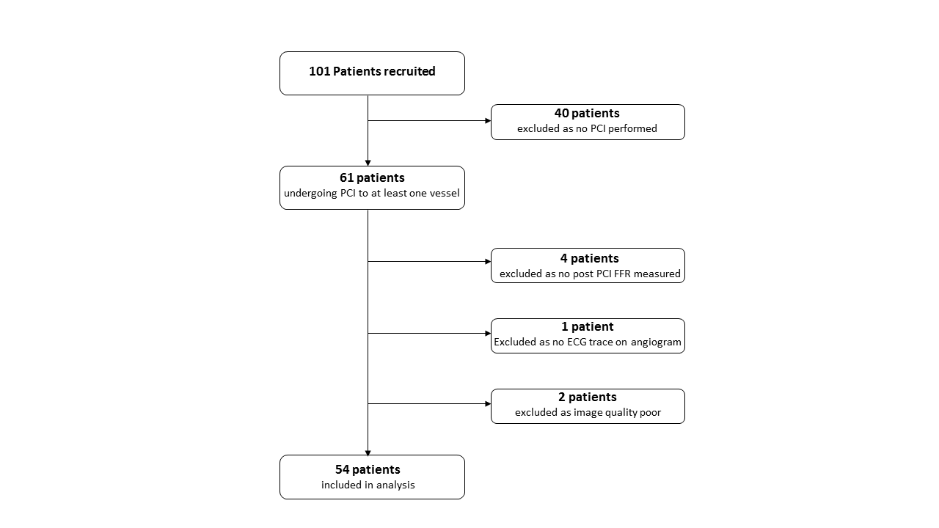
**

**Online Figure 2: Correlation between vFFR and QCA**

Scatter plot of quantitative coronary angiography (QCA) versus vFFR (left). Two cases with similar FFR values are shown in more detail (right). The left panel is an angiographic frame demonstrating the lesion. The central and right frames are two orthogonal views with QCA calculated for each view of the lesion. The final QCA value is the average of the two figures. In case 1, the FFR was 0.44 and vFFR 0.54 indicating a severe stenosis, but QCA suggested only a 44% average diameter stenosis. In case 2, the FFR was 0.45 and vFFR 0.47, again indicating a severe stenosis. In this case, QCA suggested an 83% average diameter stenosis. This highlights how QCA can vary significantly for cases with similar FFR values.

**
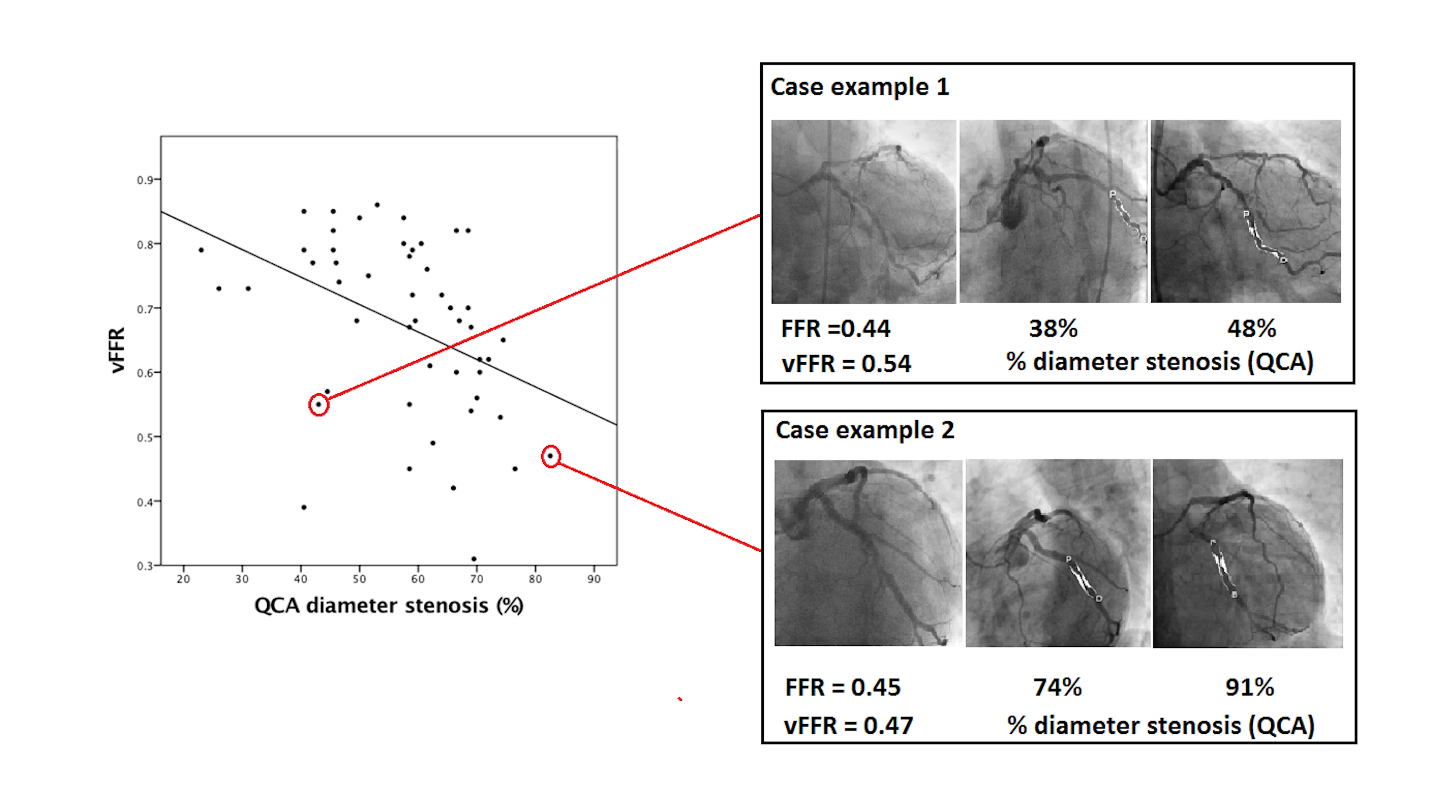
**

**Table 1: Corresponding FFR and vFFR values in all vessels studied**

|  |  | Pre-PCI | | Post-PCI/VCI | |
| --- | --- | --- | --- | --- | --- |
| Case | **Vessel** | **FFR** | **vFFR** | **FFR** | **vFFR** |
| 1 | LAD | 0.64 | 0.62 | 0.93 | 0.93 |
| 2 | RCA | 0.44 | 0.45 | 0.98 | 0.98 |
| 3 | LAD | 0.62 | 0.72 | 0.84 | 0.84 |
|  | LCX | 0.67 | 0.62 | 0.93 | 0.93 |
| 4 | RCA | 0.79 | 0.79 | 0.99 | 0.99 |
| 5 | RCA | 0.86 | 0.86 | 0.88 | 0.96 |
| 6 | LAD | 0.80 | 0.85 | 0.91 | 0.91 |
| 7 | LAD | 0.34 | 0.39 | 0.93 | 0.93 |
| 8 | LAD | 0.74 | 0.78 | 0.90 | 0.93 |
|  | LAD | 0.76 | 0.71 | 0.91 | 0.91 |
| 9 | LAD | 0.79 | 0.79 | 0.86 | 0.86 |
|  | RCA | 0.76 | 0.76 | 0.89 | 0.89 |
| 10 | LAD | 0.72 | 0.77 | 0.84 | 0.86 |
| 11 | LAD | 0.74 | 0.72 | 0.94 | 0.96 |
| 12 | RCA | 0.38 | 0.42 | 0.88 | 0.93 |
| 13 | LAD | 0.82 | 0.85 | 0.88 | 0.91 |
| 14 | LAD | 0.75 | 0.74 | 0.90 | 0.90 |
| 15 | LCX | 0.42 | 0.53 | 0.98 | 0.99 |
| 16 | RCA | 0.63 | 0.57 | 0.81 | 0.86 |
| 17 | LAD | 0.56 | 0.62 | 0.85 | 0.91 |
| 18 | LAD | 0.59 | 0.67 | 0.95 | 0.95 |
| 19 | LCX | 0.44 | 0.55 | 0.88 | 0.93 |
| 20 | LAD | 0.77 | 0.75 | 0.88 | 0.88 |
| 21 | RCA | 0.79 | 0.79 | 0.94 | 0.91 |
| 22 | LCX | 0.75 | 0.68 | 0.89 | 0.80 |
|  | RCA | 0.65 | 0.65 | 0.93 | 0.93 |
| 23 | LAD | 0.83 | 0.84 | 0.88 | 0.88 |
| 24 | RCA | 0.79 | 0.79 | 0.89 | 0.89 |
| 25 | LCX | 0.64 | 0.45 | 0.96 | 0.92 |
| 26 | LAD | 0.75 | 0.77 | 0.82 | 0.88 |
|  | RCA | 0.47 | 0.56 | 0.90 | 0.92 |
| 27 | LCX | 0.38 | 0.31 | 0.97 | 0.97 |
| 28 | LAD | 0.71 | 0.70 | 0.89 | 0.95 |
| 29 | LAD | 0.43 | 0.54 | 0.81 | 0.91 |
| 30 | LAD | 0.70 | 0.60 | 0.84 | 0.89 |
| 31 | RCA |  |  | 0.89 | 0.94 |
| 32 | LAD | 0.72 | 0.77 | 0.86 | 0.93 |
| 33 | RCA | 0.85 | 0.86 | 1 | 0.98 |
| 34 | RCA | 0.79 | 0.75 | 0.93 | 0.94 |
| 35 | LCX | 0.45 | 0.47 | 1 | 0.99 |
| 36 | LAD | 0.53 | 0.70 | 0.91 | 0.93 |
|  | LAD |  |  | 0.94 | 0.94 |
| 37 | RCA | 0.57 | 0.68 | 0.92 | 0.93 |
| 38 | LAD | 0.80 | 0.80 | 0.93 | 0.95 |
| 39 | RCA | 0.48 | 0.49 | 0.88 | 0.88 |
| 40 | RCA | 0.78 | 0.82 | 0.97 | 0.99 |
| 41 | LAD | 0.82 | 0.82 | 0.89 | 0.90 |
| 42 | RCA | 0.62 | 0.55 | 0.96 | 0.94 |
| 43 | LAD | 0.68 | 0.84 | 0.95 | 0.97 |
| 44 | LAD | 0.71 | 0.80 | 0.87 | 0.94 |
| 45 | RCA | 0.73 | 0.68 | 0.96 | 0.95 |
| 46 | LAD | 0.65 | 0.61 | 0.78 | 0.79 |
| 47 | LAD | 0.79 | 0.82 | 0.94 | 0.94 |
| 48 | LAD | 0.63 | 0.73 | 0.81 | 0.81 |
| 49 | LAD | 0.55 | 0.67 | 0.87 | 0.87 |
| 50 | LAD | 0.74 | 0.73 | 0.82 | 0.82 |
| 51 | RCA | 0.61 | 0.65 | 0.88 | 0.92 |
| 52 | RCA | 0.68 | 0.68 | 0.85 | 0.88 |
| 53 | LAD | 0.68 | 0.60 | 0.91 | 0.95 |
| 54 | RCA | 0.78 | 0.70 | 0.90 | 0.90 |

LAD = Left anterior descending, LCX = left circumflex, RCA = Right coronary artery.
